# Dichlorido(4,5-diaza­fluoren-9-one-κ^2^ *N*,*N*′)palladium(II)

**DOI:** 10.1107/S1600536809035272

**Published:** 2009-09-05

**Authors:** Zhi-Guang Xu, Hai-Yang Liu, Qing-Guang Zhan, Jian Chen, Min-Jian Xu

**Affiliations:** a School of Chemistry and Environment, South China Normal University, Guangzhou 510006, People’s Republic of China; b Department of Chemistry, South China University of Technology, Guangzhou 510641, People’s Republic of China

## Abstract

The structure of the title compound, [PdCl_2_(C_11_H_6_N_2_O)], shows a nearly square-planar geometry for the Pd^II^ atom within a Cl_2_N_2_ donor set.

## Related literature

For related palladium complexes, see: Klein *et al.* (1998 ▶).
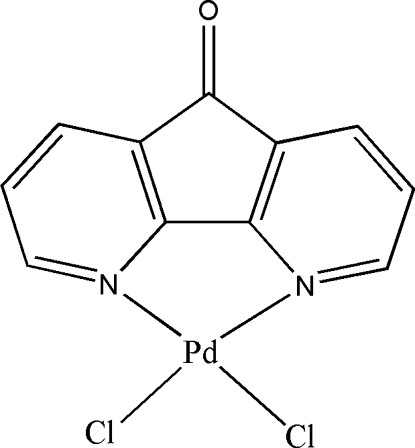


## Experimental

### 

#### Crystal data

[PdCl_2_(C_11_H_6_N_2_O)]
*M* *_r_* = 359.48
Monoclinic, 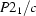

*a* = 5.131 (5) Å
*b* = 17.105 (5) Å
*c* = 12.763 (5) Å
β = 99.183 (5)°
*V* = 1105.8 (12) Å^3^
*Z* = 4
Mo *K*α radiation
μ = 2.14 mm^−1^
*T* = 293 K
0.35 × 0.33 × 0.25 mm

#### Data collection

Bruker SMART APEXII diffractometer
Absorption correction: multi-scan (*SADABS*; Bruker, 2005 ▶) *T*_min_ = 0.477, *T*_max_ = 0.586
6785 measured reflections
2703 independent reflections
2198 reflections with *I* > 2σ(*I*)
*R*_int_ = 0.028

#### Refinement

*R*[*F* ^2^ > 2σ(*F* ^2^)] = 0.029
*wR*(*F* ^2^) = 0.058
*S* = 1.04
2703 reflections
178 parameters
All H-atom parameters refined
Δρ_max_ = 0.37 e Å^−3^
Δρ_min_ = −0.54 e Å^−3^

### 

Data collection: *APEX2* (Bruker, 2005 ▶); cell refinement: *SAINT* (Bruker, 2005 ▶); data reduction: *SAINT*; program(s) used to solve structure: *SHELXS97* (Sheldrick, 2008 ▶); program(s) used to refine structure: *SHELXL97* (Sheldrick, 2008 ▶); molecular graphics: *ORTEP-3 for Windows* (Farrugia, 1997 ▶); software used to prepare material for publication: *SHELXTL* (Sheldrick, 2008 ▶).

## Supplementary Material

Crystal structure: contains datablocks I, global. DOI: 10.1107/S1600536809035272/tk2532sup1.cif

Structure factors: contains datablocks I. DOI: 10.1107/S1600536809035272/tk2532Isup2.hkl

Additional supplementary materials:  crystallographic information; 3D view; checkCIF report

## Figures and Tables

**Fig. 1.**
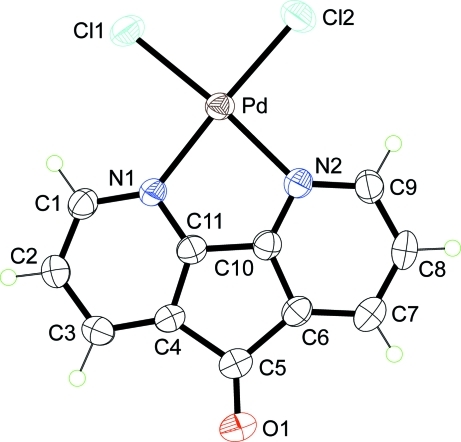
Molecular structure of (I) with displacement ellipsoids drawn at the 50% probability level.

## supplementary crystallographic information

### Comment

The title compound, (I), Fig. 1, shows the palladium atom to be coordinated by two chlorides and two nitrogen atoms, the latter derived from a chelating 4,5-diazafluoren-9-one (dafo) ligand. The coordination geometry is based on a square planar arrangement of the four donor atoms. Related palladium structures featuring dafo ligands are known (Klein *et al.*,  1998).

### Experimental

Compound (I) was synthesized hydrothermally from an aqueous mixture (10.0 ml) containing PdCl_2_ (0.1758 g) and 4,5-diazafluoren-9-one (0.301 g) in a 30 ml Teflon-lined stainless steel vessel. In the vessel, the solution was heated to 413 K for 72 h then cooled to room temperature at a rate of 10 K per hour. Brown prisms were obtained after washed thoroughly with deionized water and air-drying.

### Refinement

All H atoms were refined: range of C-H = 0.89 (3) to 0.99 (4) Å.

### Crystal data

**Table:**

[PdCl_2_(C_11_H_6_N_2_O)]	*F*(000) = 696
*M**_r_* = 359.48	*D*_x_ = 2.159 Mg m^−^^3^
Monoclinic, *P*2_1_/*c*	Mo *K*α radiation, λ = 0.71069 Å
Hall symbol: -P 2ybc	Cell parameters from 13853 reflections
*a* = 5.131 (5) Å	θ = 1.8–28.2°
*b* = 17.105 (5) Å	µ = 2.14 mm^−^^1^
*c* = 12.763 (5) Å	*T* = 293 K
β = 99.183 (5)°	Block, yellow
*V* = 1105.8 (12) Å^3^	0.35 × 0.33 × 0.25 mm
*Z* = 4	

### Data collection

**Table:**

Bruker SMART APEXII CCD area-detector diffractometer	2703 independent reflections
Radiation source: fine-focus sealed tube	2198 reflections with *I* > 2σ(*I*)
graphite	*R*_int_ = 0.028
Detector resolution: 10 pixels mm^-1^	θ_max_ = 28.4°, θ_min_ = 2.0°
ω scans	*h* = −6→6
Absorption correction: multi-scan (*SADABS*; Bruker, 2005)	*k* = −22→13
*T*_min_ = 0.477, *T*_max_ = 0.586	*l* = −16→16
6785 measured reflections	

### Refinement

**Table:**

Refinement on *F*^2^	Primary atom site location: structure-invariant direct methods
Least-squares matrix: full	Secondary atom site location: difference Fourier map
*R*[*F*^2^ > 2σ(*F*^2^)] = 0.029	Hydrogen site location: inferred from neighbouring sites
*wR*(*F*^2^) = 0.058	All H-atom parameters refined
*S* = 1.04	*w* = 1/[σ^2^(*F*_o_^2^) + (0.0199*P*)^2^ + 0.6053*P*] where *P* = (*F*_o_^2^ + 2*F*_c_^2^)/3
2703 reflections	(Δ/σ)_max_ < 0.001
178 parameters	Δρ_max_ = 0.37 e Å^−^^3^
0 restraints	Δρ_min_ = −0.54 e Å^−^^3^

### Special details

**Table:**

Geometry. All e.s.d.'s (except the e.s.d. in the dihedral angle between two l.s. planes) are estimated using the full covariance matrix. The cell e.s.d.'s are taken into account individually in the estimation of e.s.d.'s in distances, angles and torsion angles; correlations between e.s.d.'s in cell parameters are only used when they are defined by crystal symmetry. An approximate (isotropic) treatment of cell e.s.d.'s is used for estimating e.s.d.'s involving l.s. planes.
Refinement. Refinement of *F^2^* against ALL reflections. The weighted *R*-factor *wR* and goodness of fit *S* are based on *F^2^*, conventional *R*-factors *R* are based on *F*, with *F* set to zero for negative *F^2^*. The threshold expression of *F^2^* > σ(*F^2^*) is used only for calculating *R*factors(gt) *etc*. and is not relevant to the choice of reflections for refinement. *R*-factors based on *F^2^* are statistically about twice as large as those based on *F*, and *R*-factors based on ALL data will be even larger.

### Fractional atomic coordinates and isotropic or equivalent isotropic displacement parameters (Å^2^)

**Table:**

	*x*	*y*	*z*	*U*_iso_*/*U*_eq_	
Pd1	0.08571 (4)	0.152850 (13)	0.283179 (18)	0.03075 (8)	
N2	0.2819 (5)	0.16090 (14)	0.15176 (19)	0.0336 (6)	
N1	0.2865 (4)	0.04745 (13)	0.30833 (19)	0.0303 (5)	
C6	0.6231 (5)	0.08217 (18)	0.0892 (2)	0.0343 (7)	
C4	0.6221 (6)	−0.01682 (17)	0.2253 (2)	0.0322 (6)	
C5	0.7568 (6)	0.00609 (19)	0.1320 (2)	0.0378 (7)	
O1	0.9357 (5)	−0.02803 (14)	0.10159 (19)	0.0532 (6)	
C11	0.4449 (5)	0.04170 (17)	0.2364 (2)	0.0319 (6)	
C10	0.4439 (5)	0.10020 (17)	0.1557 (2)	0.0317 (6)	
C3	0.6398 (6)	−0.0784 (2)	0.2962 (3)	0.0402 (8)	
C8	0.4677 (7)	0.1976 (2)	−0.0016 (3)	0.0434 (8)	
C1	0.3028 (6)	−0.01242 (19)	0.3780 (3)	0.0367 (7)	
C9	0.2942 (6)	0.2103 (2)	0.0695 (3)	0.0397 (7)	
C7	0.6379 (7)	0.1337 (2)	0.0064 (3)	0.0414 (8)	
C2	0.4742 (6)	−0.0747 (2)	0.3724 (3)	0.0413 (8)	
Cl1	−0.10542 (16)	0.13410 (5)	0.42956 (7)	0.0445 (2)	
Cl2	−0.11747 (16)	0.26981 (5)	0.24949 (7)	0.0479 (2)	
H1	0.187 (6)	−0.0098 (17)	0.429 (2)	0.038 (9)*	
H3	0.762 (7)	−0.123 (2)	0.295 (3)	0.052 (10)*	
H4	0.753 (6)	0.1289 (19)	−0.038 (3)	0.048 (10)*	
H2	0.477 (6)	−0.115 (2)	0.420 (3)	0.046 (9)*	
H5	0.465 (6)	0.2338 (19)	−0.055 (3)	0.043 (9)*	
H6	0.181 (6)	0.2543 (19)	0.064 (2)	0.046 (9)*	

### Atomic displacement parameters (Å^2^)

**Table:**

	*U*^11^	*U*^22^	*U*^33^	*U*^12^	*U*^13^	*U*^23^
Pd1	0.02879 (12)	0.02863 (13)	0.03619 (14)	−0.00123 (9)	0.00940 (9)	−0.00117 (10)
N2	0.0321 (13)	0.0332 (14)	0.0364 (14)	−0.0019 (11)	0.0084 (10)	0.0016 (11)
N1	0.0293 (12)	0.0303 (13)	0.0328 (14)	−0.0010 (10)	0.0097 (10)	0.0015 (10)
C6	0.0300 (15)	0.0392 (18)	0.0341 (17)	−0.0042 (13)	0.0070 (12)	−0.0008 (13)
C4	0.0306 (15)	0.0341 (16)	0.0330 (17)	−0.0017 (12)	0.0086 (12)	−0.0034 (13)
C5	0.0351 (16)	0.0419 (18)	0.0373 (18)	−0.0013 (14)	0.0093 (14)	−0.0042 (14)
O1	0.0540 (14)	0.0596 (16)	0.0516 (15)	0.0154 (12)	0.0258 (12)	0.0029 (12)
C11	0.0281 (15)	0.0337 (16)	0.0340 (17)	−0.0032 (12)	0.0056 (12)	−0.0019 (12)
C10	0.0279 (14)	0.0343 (17)	0.0329 (16)	−0.0027 (12)	0.0049 (12)	0.0010 (13)
C3	0.0390 (18)	0.0366 (18)	0.046 (2)	0.0071 (14)	0.0092 (15)	0.0012 (15)
C8	0.051 (2)	0.045 (2)	0.0340 (19)	−0.0075 (16)	0.0075 (15)	0.0118 (15)
C1	0.0366 (17)	0.0387 (18)	0.0366 (18)	−0.0027 (14)	0.0112 (14)	0.0016 (14)
C9	0.0401 (18)	0.0362 (18)	0.043 (2)	0.0004 (15)	0.0070 (14)	0.0079 (14)
C7	0.0427 (19)	0.049 (2)	0.0345 (19)	−0.0075 (15)	0.0114 (15)	−0.0019 (15)
C2	0.0461 (19)	0.0348 (18)	0.044 (2)	0.0026 (15)	0.0122 (15)	0.0084 (15)
Cl1	0.0490 (5)	0.0435 (5)	0.0461 (5)	−0.0033 (4)	0.0235 (4)	−0.0035 (4)
Cl2	0.0470 (5)	0.0334 (4)	0.0651 (6)	0.0065 (4)	0.0147 (4)	0.0033 (4)

### Geometric parameters (Å, °)

**Table:**

Pd1—N1	2.076 (2)	C4—C5	1.521 (4)
Pd1—N2	2.094 (3)	C5—O1	1.203 (4)
Pd1—Cl2	2.2652 (11)	C11—C10	1.436 (4)
Pd1—Cl1	2.2672 (12)	C3—C2	1.391 (5)
N2—C10	1.326 (4)	C8—C9	1.387 (5)
N2—C9	1.356 (4)	C8—C7	1.392 (5)
N1—C11	1.323 (4)	C1—C2	1.390 (5)
N1—C1	1.350 (4)	H3—C3	0.99 (4)
C6—C10	1.382 (4)	H1—C1	0.95 (3)
C6—C7	1.387 (4)	H2—C2	0.92 (4)
C6—C5	1.531 (4)	H5—C8	0.92 (4)
C4—C11	1.375 (4)	H6—C9	0.95 (3)
C4—C3	1.382 (4)	H4—C7	0.89 (3)
			
N1—Pd1—N2	83.74 (10)	C4—C11—C10	111.1 (3)
N1—Pd1—Cl2	176.78 (7)	N2—C10—C6	128.8 (3)
N2—Pd1—Cl2	93.17 (8)	N2—C10—C11	120.2 (3)
N1—Pd1—Cl1	91.09 (7)	C6—C10—C11	111.0 (3)
N2—Pd1—Cl1	174.80 (7)	C4—C3—C2	116.1 (3)
Cl2—Pd1—Cl1	92.00 (4)	C9—C8—C7	122.3 (3)
C10—N2—C9	114.2 (3)	N1—C1—C2	121.3 (3)
C10—N2—Pd1	107.26 (19)	N2—C9—C8	121.6 (3)
C9—N2—Pd1	138.5 (2)	C6—C7—C8	116.4 (3)
C11—N1—C1	114.8 (3)	C1—C2—C3	122.2 (3)
C11—N1—Pd1	107.58 (19)	C7—C8—H5	121.(2)
C1—N1—Pd1	137.5 (2)	C9—C8—H5	116.(2)
C10—C6—C7	116.7 (3)	N1—C1—H1	115.8 (18)
C10—C6—C5	106.0 (3)	C2—C1—H1	122.8 (18)
C7—C6—C5	137.4 (3)	C1—C2—H2	119.(2)
C11—C4—C3	117.6 (3)	C3—C2—H2	119.(2)
C11—C4—C5	106.5 (3)	N2—C9—H6	116.7 (17)
C3—C4—C5	135.9 (3)	C8—C9—H6	121.8 (17)
O1—C5—C4	126.4 (3)	C4—C3—H3	124.(2)
O1—C5—C6	128.2 (3)	C2—C3—H3	120.(2)
C4—C5—C6	105.3 (2)	C6—C7—H4	124.(2)
N1—C11—C4	128.0 (3)	C8—C7—H4	120.(2)
N1—C11—C10	120.9 (3)		
